# Who Makes it Home: SNF to Community Transitions for Traditional Medicare Beneficiaries with Serious Mental Illness

**DOI:** 10.1093/geroni/igaf122.014

**Published:** 2025-12-31

**Authors:** Taylor Bucy, Carrie Henning-Smith, Donovan Maust, Tetyana Shippee, Dori Cross

**Affiliations:** University of Kansas School of Medicine, Kansas City, Kansas, United States; University of Minnesota School of Public Health, Minneapolis, Minnesota, United States; University of Michigan Medical School, Ann Arbor, Michigan, United States; University of Minnesota, Minneapolis, Minnesota, United States; University of Minnesota School of Public Health, Minneapolis, Minnesota, United States

## Abstract

Discharge to the community following a skilled nursing facility (SNF) stay is a key metric of high-quality care. This aligns with preferences around aging-in-place, yet significant fragmentation across service providers has made achieving this outcome challenging. This is especially true for people with serious mental illness (SMI) (i.e. schizophrenia, bipolar disorder) who represent a growing proportion of U.S. long-term care residents. We use a 4-year (2016-2019), 100% sample of traditional Medicare data to examine individual- and organization-level predictors of SNF discharge location (community vs other) for persons with SMI. Of the 118,325 unique SNF stays for people with SMI, 54% ended in discharge to the community. Patients with SMI who were discharged to the community (vs not) were significantly younger (69 vs 72 y/o), more likely to be female (61% vs 56%), and were less likely to be dual-eligible (51% vs 71%) or to have co-occurring ADRD (33% vs 51%). SMI patients discharged to the community were also significantly more likely to receive care in smaller, urban SNFs, that were more integrated, higher quality, and saw a smaller share of SMI patients overall. These findings were reinforced by our fully adjusted regression models. While many socioenvironmental factors restrict patients’ ability to return home following a SNF stay, better positioning SNFs to achieve good outcomes requires significant investments along the continuum. Policymakers should consider how to leverage value-based payment programs in a way that better accounts for the resources required to coordinate care for this population, including partnership with community-based providers.

